# Pediatric neurobrucellosis and atypical Guillain-Barré syndrome: an intriguing case unveiled

**DOI:** 10.25122/jml-2023-0522

**Published:** 2024-08

**Authors:** Huda Zaher, Ahmed Bamaga, Suzan Alshihri

**Affiliations:** 1 Internal Medicine Department, Neurology Division, King Fahad General Hospital, Jeddah, Saudi Arabia; 2 College of Medicine, King Abdulaziz University, Jeddah, Saudi Arabia; 3 Pediatric Department, Neurology Division, King Abdulaziz University Hospital, Jeddah, Saudi Arabia

**Keywords:** Brucella, neurobrucellosis, polyradiculoneuropathy, meningitis

## Abstract

Brucellosis, a chronic zoonotic disease with a significant global burden, particularly in endemic areas, can also present as neurobrucellosis, a rare complication. We report a case of polyradiculoneuropathy in a pediatric patient resulting from this uncommon presentation. A 5-year-old girl presented with progressive asymmetric lower limb weakness for two weeks that progressed to a loss of ambulation in four weeks. She also had flu-like symptoms and persistent high-grade fever. Her history was notable for ingesting raw milk before the onset of fever. Initial examination revealed meningismus signs and fever. She had bilateral weak hip flexion graded 4/5 on the Medical Research Council (MRC) scale, weak right and left knee flexion (3/5 and 4/5, respectively), and weak right ankle dorsiflexion (4/5). She also had diminished reflexes throughout. The patient exhibited a clinical picture resembling Guillain-Barré Syndrome (GBS) after admission as her weakness progressed. Cerebrospinal fluid (CSF) analysis revealed no cell counts, elevated protein levels (1545 mg/dL), and normal glucose levels (3.34 mmol/L). Blood and CSF cultures were negative, but the serum antibody titer was elevated at 1:1280 against *Brucella melitensis* and *Brucella abortus* species. Lumbosacral MRI showed diffuse enhancement of the lower nerve roots. A nerve conduction study (NCS) demonstrated axonal and demyelinating polyradiculoneuropathy. The patient regained her strength three months after presentation, following a course of antibiotics. When evaluating patients with atypical manifestations resembling GBS, brucellosis should be considered an important differential diagnosis in endemic areas.

## INTRODUCTION

Brucellosis is a chronic granulomatous zoonotic disease caused by *Brucella* species [1]. Neurobrucellosis is a significant burden that affects patients worldwide, particularly in endemic areas [2]. High-risk regions include Mexico, South and Central America, countries around the Mediterranean Sea, and the Middle East [3]. Transmission to humans occurs through ingesting infected, unpasteurized animal milk products or inhaling aerosolized infected particles [1]. Brucellosis can present with various manifestations, such as osteoarticular complaints, hepatitis, or neurological symptoms [1]. Neurological manifestations commonly seen in acute infection include headaches, myalgia, and fatigue [4-6]. Central nervous system (CNS) involvement is found in 10% of the cases [7]. Meningoencephalitis, brain abscesses, and demyelinating syndromes have all been reported [7]. Approximately 7% of neurobrucellosis cases affect the peripheral nervous system (PNS), presenting as either chronic polyradiculoneuritis or acute neuropathy [8,9]. Guillain-Barré syndrome (GBS) is a polyradiculoneuropathy that typically peaks in severity within four weeks. It presents with a rapid or more gradual onset of relatively symmetrical weakness and reduced or absent reflexes, coupled with albuminocytological dissociation in the cerebrospinal fluid (CSF). GBS is thought to be an immune response-related disorder linked to various infectious agents, including *Cytomegalovirus, Epstein-Barr* virus, *Campylobacter jejuni*, and *Mycoplasma pneumonia* [10]. Nonetheless, distinguishing between neuropathies caused by infectious agents and those arising as post-infectious, immune-mediated conditions like GBS can be complex [11]. Such diagnostic challenges may raise uncertainty on the infrequently suggested link between GBS and brucellosis. In this case report, we present a rare occurrence of PNS involvement mimicking GBS due to brucellosis infection in a pediatric patient.

## CASE PRESENTATION

A 5-year-old girl presented to the emergency room with a 2-week history of progressive walking difficulty. She initially noted some heaviness and pain in her lower limbs. Over time, she developed asymmetric lower limb weakness that started in her right lower limb and then involved the left one, causing unsteadiness while standing and walking and eventually leading to the loss of ambulation in four weeks. She denied any changes in sensation or strength in her upper limbs. Two weeks before these walking problems began, she experienced flu-like symptoms: persistent high-grade fever, sweating, chills, myalgia, and neck and back pain. She denied a history of diplopia or ocular or facial weakness. She had no symptoms related to sphincter control or cognitive dysfunction. She had no other systemic manifestations, including cardiorespiratory symptoms or gastrointestinal complaints. She is the first child of consanguineous parents with no family history of any similar condition or neurological disorders. She had a normal developmental background, and her history was significant only for consuming raw camel milk before the onset of fever.

At presentation, the patient was fully conscious but lethargic. Vital signs showed a high-grade fever of 38.1°C, but the other parameters were stable. She had positive meningismus signs, including Kernig's and Brudzinski's signs. She showed intact cognitive and cranial nerve functions. The muscle bulk and tone were preserved throughout. Her strength was normal in neck flexion and the upper limbs. However, she had asymmetric weakness in her lower limbs with the following Medical Research Council (MRC) grades: bilateral hip flexion at 4, right knee flexion at 3, left knee flexion at 4, and right ankle dorsiflexion at 4. Strength in the remaining muscle groups of the lower limbs was normal.

She had bilaterally diminished deep tendon reflexes in the biceps, triceps, brachioradialis, patellar, and Achilles tendons. She had a flexor plantar response. Her sensory examination was intact, including fine touch, pinprick, temperature, vibration, and proprioception. Additionally, the cerebellar examination showed no abnormalities. A wide-based gait was observed with a negative Romberg sign and absent scapular winging. No neurocutaneous stigmata or dysmorphic features were observed. Systemic examinations were unremarkable, with no signs of respiratory distress or hepatosplenomegaly.

**Figure 1:**
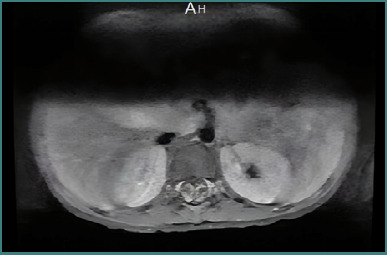
Axial T1-weighted gadolinium-enhanced MRI of the lumbar spine showing cauda equina nerve roots enhancement

The investigations showed normal results for the complete blood count, basic metabolic panel (including creatinine, urea, magnesium, phosphate, and calcium), and liver function test. The levels of thyroid-stimulating hormone, thyroxine, and vitamin D were also within the normal range. Serum creatinine kinase (CK) levels were normal at 37 U/L. However, the erythrocyte sedimentation rate (ESR) was elevated at 18 mm/h. Antinuclear antibodies (ANA) and double-stranded DNA tests came back negative. Blood bacterial cultures were obtained but showed no growth. The serum *Brucella* antibody titer was elevated at 1:1280, positive for both *Brucella melitensis* and *Brucella abortus*. A lumbar puncture was performed, and the analysis of CSF revealed no cell counts, elevated protein levels (1545 mg/dL), and normal glucose levels (3.34 mmol/L). Bacterial and fungal cultures of CSF were negative. A lumbosacral spine MRI with contrast showed enhancement of the lower spinal cord meninges and thickening and enhancement of the cauda equine nerve roots (Figure 1). A nerve conduction study (NCS) showed evidence of mixed axonal and demyelinating polyradiculoneuropathy (Table 1).

**Table 1:** Nerve conduction study

Motor nerves	DL (ms)	Amp (mV)	CV (m/s)	F waves (ms)
**Right median**				
Wrist-APB	9.15	2.2		43.0
Elbow-Wrist	14.5	1.30	20.6	
**Right ulnar**				
Wrist-ADM	7.39	1.68		43.7
Below elbow-Wrist	11.4	0.88	24.9	
Above elbow-below elbow	13.8	0.69	25.0	
**Right tibialis**				
Ankle-Abd hal	6.62	0.55		64.7
Knee-Ankle	16.0	0.53	23.5	
**Right peroneal**				
Ankle-EDB	10.7	0.58		67.9
Below knee-ankle	16.8	0.35	31.1	
Fibular head-below knee	18.0	0.35	33.3	
**Sensory nerves**	**DL (ms)**	**Amp (µV)**	**CV (m/s)**	
**Right median**				
Digit II-Wrist	4.07	8.6	28.3	
Palm-Wrist	2.73	10.5	32.4	
**Right ulnar**				
Digit IV-Wrist	2.49	0.99	33.6	
Digit V-Wrist	3.90	5.5	34.0	
**Right radial**				
EPL tendon-Wrist	2.96	18.1	45.7	
**Right sural**				
Mid lower leg-Lateral malleolus	4.90	4.9	Not recordable	

Amp, amplitude; CV, conduction velocity; DL, distal latency; ms, milliseconds; mV, millivolts; µV, microvolts; m/s, meter per second

Based on these results, the patient was started on a two-week course of amikacin and a six-week course of ceftriaxone. A four-month course of rifampicin and Bactrim was planned. Intravenous (IV) methylprednisolone was given for two weeks and then tapered down orally over two weeks. Additionally, the patient was prescribed gabapentin for neuropathic pain, as well as vitamins B6 and B12. Physiotherapy was also recommended as part of the treatment plan. The patient successfully regained her full strength three months after her initial presentation.

## DISCUSSION

This case presents an acute to subacute onset of multifocal localization of meningismus irritation and sensorimotor deficits that notably affect the lower limbs. The patient’s lower limb weakness involved both proximal and distal muscles asymmetrically, back and leg pain, and diffuse hyporeflexia. These findings indicate a lower motor neuron lesion and suggest a sensorimotor PNS pathology. These findings may also indicate a leptomeningeal process, given the febrile illness and positive meningismus signs during the initial presentation. Possible lumbosacral polyradiculoneuropathy, along with meningismus irritation, suggests leptomeningeal involvement of both the brain and spinal cord.

As a general approach, the differential diagnosis for these acute to subacute neurological manifestations is generally broad and includes metabolic, inflammatory, infectious, and neoplastic processes. Examples of metabolic disorders are diabetes, porphyria, and vitamin deficiencies. Inflammatory conditions may include GBS, neurosarcoidosis, systemic lupus erythematosus, and Sjögren's syndrome. Infectious causes may involve fungal or bacterial etiologies such as cryptococcal infection, tuberculosis, or brucellosis. Examples of neoplastic processes are leptomeningeal carcinomatosis and lymphoma.

In our case presentation, the patient was initially brought to the hospital due to febrile illness and lethargy, along with neck stiffness and lower limb weakness. The clinical presentation at first raised suspicions of meningitis, prompting us to perform a lumbar puncture and obtain CSF samples for analysis. Her CSF study showed no cell counts, high protein, and normal glucose. Further workup confirmed the causative etiology, leading to a final diagnosis of neurobrucellosis as confirmed by the high *Brucella* antibody titer. The patient was promptly started on an antibiotic regimen. However, after the second week of admission, she had a progressive weakness in her lower limbs, resulting in a loss of ambulation as her muscle strength became 2 to 3 out of 5. Notably, there was no facial, upper limbs, or neck flexor weakness or symptoms and signs suggesting respiratory muscle involvement. Given the progression of her symptoms after her initial flu-like symptoms, GBS was highly suspected. As a result, an MRI of the lower spine with contrast was performed, showing enhancement of the lumbosacral nerve roots and spinal meninges. Additionally, the NCS revealed evidence of mixed axonal and demyelinating polyneuropathy. We monitored her closely for more than a week to track any potential exacerbation of her symptoms. Fortunately, she began showing slight improvements in her lower limb strength as her antibiotic treatment continued, and her weakness did not progress further. Based on these observations, we related that her GBS-like symptoms were atypical and were likely linked to the primary brucellosis infection.

During the active phase of GBS, we suspected a connection to brucellosis. This suspicion was supported by laboratory diagnostic criteria, including a positive blood culture for *Brucella*, the presence of *Brucella* species in the cerebrospinal fluid, the detection of *Brucella* DNA using polymerase chain reaction (PCR), or elevated levels of *Brucella*-specific antibodies in either the blood or CSF [12].

A few studies have been published on the involvement of PNS in brucellosis infections. Most of these are case reports. A study by Gulcin Benbir *et al*. [13] reported axonal sensory involvement in patients with brucellosis and mild conduction abnormalities in sensory and motor nerves. Another study focused on peripheral neuropathy caused by brucellosis in 34 patients; 12 (35.3%) had sensorimotor axonal peripheral neuropathy [14]. In 2009, Kaya *et al*. [15] described a case of brucellosis with a macular rash and peripheral neuropathy; electrophysiological investigation indicated mild demyelinating sensory polyneuropathy. Additionally, Al-Deeb *et al*. [5] reported 13 patients with neurobrucellosis, 3 of whom had PNS impairment — 2 with mononeuritis and 1 with demyelinating polyradiculopathy [5].

The pathogenesis of PNS involvement in brucellosis is not yet fully understood. The organisms are believed to be able to survive intracellularly within phagocytes, potentially leading to PNS pathology [5]. The involvement may occur through direct invasion of the organism and endotoxins into the peripheral nerves, causing axonal polyneuropathy, or via immune-mediated mechanisms that lead to demyelination [5].

Less than half of brucellosis patients with neurological manifestations show abnormal imaging findings [14,15]. Neuroimaging may reveal the involvement of the meninges, vasculature, cranial nerves, and spinal nerve roots [16,17].

The relapse rate is estimated at 5% to 10% [18]. Therefore, a prolonged course of treatment with an antibiotic with strong cell wall permeability and CNS penetration is recommended [19]. The standard treatment regimens include doxycycline, rifampin, and ceftriaxone [20]. Quinolones can be administered to patients allergic to cephalosporins [19]. The treatment should be continued for at least six weeks [21]. The use of prednisolone is also advised to prevent the long-term complications associated with neurobrucellosis [2,7].

## CONCLUSION

This case highlights peripheral nervous system involvement in neurobrucellosis as a sequel of the leptomeningeal process. While the connection between GBS and brucellosis infection remains to be established, clinicians should consider brucellosis as a possible cause in regions where it is common, especially when patients exhibit symptoms unusual for GBS.

## Personal thanks

The authors thank Scribendi (www.scribendi.com) for editing the English language.
